# The PhosphoGRID *Saccharomyces cerevisiae* protein phosphorylation site database: version 2.0 update

**DOI:** 10.1093/database/bat026

**Published:** 2013-05-11

**Authors:** Ivan Sadowski, Bobby-Joe Breitkreutz, Chris Stark, Ting-Cheng Su, Matthew Dahabieh, Sheetal Raithatha, Wendy Bernhard, Rose Oughtred, Kara Dolinski, Kris Barreto, Mike Tyers

**Affiliations:** ^1^Department of Biochemistry and Molecular Biology, Molecular Epigenetics, Life Sciences Institute, University of British Columbia, 2350 Health Sciences Mall, Vancouver, British Columbia, Canada V6T 1Z3, ^2^Samuel Lunenfeld Research Institute, 600 University Avenue, Toronto, Ontario M5G 1X5 Canada, ^3^Lewis-Sigler Institute for Integrative Genomics, Princeton University, Princeton, NJ 08544, USA and ^4^Institute for Research in Immunology and Cancer, Université de Montréal, Pavillon Marcelle-Coutu, 2950, chemin de Polytechnique, Montréal, Québec H3T 1J4, Canada

## Abstract

PhosphoGRID is an online database that curates and houses experimentally verified *in vivo* phosphorylation sites in the *Saccharomyces cerevisiae* proteome (www.phosphogrid.org). Phosphosites are annotated with specific protein kinases and/or phosphatases, along with the condition(s) under which the phosphorylation occurs and/or the effects on protein function. We report here an updated data set, including nine additional high-throughput (HTP) mass spectrometry studies. The version 2.0 data set contains information on 20 177 unique phosphorylated residues, representing a 4-fold increase from version 1.0, and includes 1614 unique phosphosites derived from focused low-throughput (LTP) studies. The overlap between HTP and LTP studies represents only ∼3% of the total unique sites, but importantly 45% of sites from LTP studies with defined function were discovered in at least two independent HTP studies. The majority of new phosphosites in this update occur on previously documented proteins, suggesting that coverage of phosphoproteins in the yeast proteome is approaching saturation. We will continue to update the PhosphoGRID data set, with the expectation that the integration of information from LTP and HTP studies will enable the development of predictive models of phosphorylation-based signaling networks.

**Database URL:** http://www.phosphogrid.org/

## Background

Protein phosphorylation is the most thoroughly documented and characterized post-translational protein modification in eukaryotes. As a result, phosphorylation and dephosphorylation of proteins at specific residues represent well-established mechanisms for regulation of protein interactions, activity, conformation and localization in response to signaling events (1). High-throughput (HTP) strategies for detection and sequence determination of phosphopeptides by mass spectrometry (MS) have lead to a massive expansion in the identification of specific phosphosites on proteins from many eukaryotic organisms. However, the biological interpretation of this information requires bioinformatic resources and tools that enable analysis of relationships between specific phosphorylation events and the causal physiological stimuli. Toward this goal, we developed PhosphoGRID as an open access database of experimentally verified *in vivo* protein phosphorylation sites focused on the model eukaryote *Saccharomyces cerevisisae* (2).

PhosphoGRID incorporates data from HTP MS phosphoproteomics studies in addition to focused low-throughput (LTP) analyses of individual proteins or complexes. Salient features of the database include documentation of each *in vivo* phosphorylation site by a defined hierarchy of experimental evidence codes, linkage of phosphorylation sites with experimentally defined protein kinases and/or phosphatases, annotation of specific condition(s) under which the phosphorylation event occurs and a description of the effect(s) that phosphorylation has on protein function. We have also documented the effects of known specific regulatory subunits for protein kinases and phosphatases, which regulate phosphorylation or dephosphorylation, respectively. Information on any substrate, protein kinase or phosphatase in PhosphoGRID can be obtained through the web-based search interface. Phosphosites are highlighted as red text on protein substrates in a sequence-based viewer. A summary of evidence for the phosphorylation event, as well as the specific condition(s) it occurs under, appears on mouse-over of the phosphosite. Consensus sequences for a limited number of protein kinases (3), which overlap verified phosphosites, are indicated in blue text on the phosphoprotein sequence. Information relating to specific protein kinases and phosphatases are also displayed in table format below the protein sequence information. All records are cross-referenced with the BioGRID interaction database (2), the *Saccharomyces* Genome Database (SGD) (4) and the NCBI protein database (5), and hyperlinks are provided to original articles in PubMED. Additionally, SGD now provides direct links to PhosphoGRID records within the protein information section of each gene summary page (6). All of the data within PhosphoGRID are freely available for download in text file format. An online submission form allows users to contribute unpublished or newly published information. Comments, corrections and/or clarifications regarding PhosphoGRID can be sent to admin@phosphogrid.org. Since its introduction in 2010, PhosphoGRID has become one of the five most visited online databases by the *Saccharomyces* research community, along with SGD (6), Yeastract (7), CYGD-MIPS (8) and BioGRID (9).

## Overview of the PhosphoGRID version 2.0 data set

The initial version of PhosphoGRID (version 1.0) included phosphosites annotated from articles published before the end of 2009, based on a standard PubMed query to retrieve candidate publications (2). PhosphoGRID version 2.0 expands the data set with results from new articles published up to June of 2011, representing an additional 190 publications, including nine articles that report HTP analysis of the budding yeast phosphoproteome by MS. The additional HTP data sets comprise analysis of rapamycin-sensitive phosphorylation (10, 11), Cdk1-dependent phosphorylation (12), phosphorylation on protein kinases, phosphatases and their associated interaction partners (13), in addition to phosphosites that accumulate during osmotic stress response (14), DNA damage (15) and perturbation of *N*-acetyltransferase expression (16) or disruption of genes encoding 124 different protein kinases and phosphatases (17). We also performed additional searches of the published yeast literature with broader terms to identify articles that may have been omitted in version 1.0; from this effort we examined an additional 305 articles in detail that were published as early as 1976. Cumulatively for PhosphoGRID versions 1.0 and 2.0, we have examined the abstracts of 5143 articles that included keywords relating to phosphorylation. Of these publications, 1008 were judged to contain potential information pertaining to phosphorylation of specific residues, and all of these were examined in full detail. Of this latter subset, 553 articles provided documentation of specific phosphorylated residues *in vivo*.

The updated data set in version 2.0 contains information on 20 178 unique phosphorylated residues, representing a 4-fold increase from version 1.0 (Table 1). The vast majority of additional unique sites are derived from the nine additional HTP studies, and the combined total number of sites from HTP analysis has increased to 19 159. Importantly, PhosphoGRID version 2.0 also contains 1614 unique phosphosites derived from LTP studies, which represents a 2-fold increase from version 1.0 (Table 1). Of these, 327 sites are recorded from articles published since the end of 2009, with an additional 437 unique LTP sites derived from articles published before 2010 that were overlooked in our initial literature search (2).

**Table 1.** Overview of the expanded data set in PhosphoGRID 2.0

PhosphoGRID version	V 1.0	V 2.0	Increase^a^
Total phosphoproteins	1495	3188	2.1
Total unique sites	4965	20 178	4.1
Total HTP sites^b^	4263	19 159	4.5
Total LTP sites^c^	851	1614	1.9
Overlap HTP/LTP^d^	149	584	3.9
% Overlap^e^	3.00	2.90	1.0

^a^Fold difference between PhospoGRID version 2.0 relative to 1.0.

^b^Total number of unique phosphosites identified from HTP MS studies.

^c^Total number of unique phosphosites identified by low LTP analysis of single proteins or protein complexes.

^d^Unique sites in common between HTP and LTP studies.

^e^The percentage of sites with overlap between the HTP and LTP data sets relative to the total number of unique sites in PhosphoGRID.

Approximately 850 total unique sites were recorded from LTP publications in version 1.0 of the PhosphoGRID (2). Many additional sites, representing 20% of the total LTP sites in version 2.0, are derived from articles published in only 15 months following the release of version 1.0. One factor that has contributed to the acceleration in documentation of phosphosites from LTP studies is the increased use of MS analysis of phosphopeptides from proteins or protein complexes purified from yeast. Analysis of phosphorylation by this strategy often results in identification of numerous phosphorylation sites, some of which may not contribute to an obvious function or phenotype. Several examples from the current update include analysis of the minichromosome maintenance (MCM) replicative helicase complex (18), the Atg1 protein kinase (19, 20) and the Sld2/Sld3 replication factors (21). An additional parameter that has contributed to prolific documentation of phosphosites in LTP studies is the production of mutants that bear multiple amino acid substitutions as a means to identify potential phosphorylated residues. Such mutants may be designed to eliminate multiple phosphorylation sites for specific protein kinases based on similarity to a consensus motif, or to eliminate a cohort of sites identified by MS or phosphorylated peptide arrays. In one such example, 38 sites of Sld3 were simultaneously eliminated to uncover a role for Rad53 in replication control (21). In many instances, however, follow-up studies to pinpoint relevant phosphorylation sites(s) have yet to be carried out, such that functional assignment is ambiguous. Nevertheless, because these results constitute evidence for phosphorylation *in vivo*, we have recorded each of the residues where mutations were made in attempts to deduce functional phosphorylation sites. For potential phosphorylation sites from these types of studies, we have included a mouse-over note on the protein sequence that describes the effect(s) produced by the multiple-site mutation. For example, a Rec8 mutant with 24 point mutations of potential casein kinase 1 (CK1) and Cdc7 kinase (Dbf4 dependent kinase (DDK)) sites eliminated detectable phosphorylation of Rec8 protein and produced a discernable phenotype (22). Each of the mutated residues on Rec8 is annotated with the statement ‘the Rec8-24A mutant protein resists removal from chromatin and degradation at the metaphase I-to-anaphase I transition and prevents conversion of bivalent chromosomes to dyads’. Similar entries were included for Rim101 (23), Slx4 (24), Zip1 (25), Red1 (26), Maf1 (27), Sld3 (21) and Dbf4 (28). Finally, several phosphoproteins, including Rpo21 and Spt5, have C-terminal domains (CTD) with multiple repeats of phosphorylated sequences. Rpo21 is the largest catalytic subunit of RNA Polymerase II and bears a CTD composed of 26 repeats with the consensus YSPTSPS. Serines 2, 5 and 7 (29–32) are known to be phosphorylated, as detected with antiphosphopeptide antibodies, but it has not been established whether each of the serines within all 26 repeats are phosphorylated *in vivo*. Similarly, the transcriptional elongation factor Spt5 has 15 C-terminal repeats with the consensus S(T/A)WGG(Q/A), on which phosphorylation has been detected by anti-phosphopeptide antibodies and MS (33). However, like the CTD of Rpo21, it has not been established whether all 15 of the serines are phosphorylated *in vivo*. Consequently, the Rpb1 and Spt5 repeat phosphorylation sites have added 41 new LTP sites to the version 2.0 update, but with the caveat that specific phosphorylation of all of these residues has not been verified.

## Comparison of LTP and HTP data sets

The overlap between phosphosites in LTP versus HTP data sets provides an important benchmark for HTP methods and facilitates the application of HTP data in hypothesis generation. In the version 1.0 data set of PhosphoGRID, we recorded 149 sites in common between the HTP and LTP data sets, which represented ∼3% of the total unique phosphosites in the database (Table 1). For the version 2.0 update, we have added ∼14 500 additional unique phosphosites from HTP studies. Given the finite number of unique sites within the yeast phosphoproteome, we expected that as the number of HTP sites increased a corresponding increase in the number of unique sites in common with the LTP data set should occur. However, despite an additional ∼800 unique sites derived from LTP studies, the number of sites in common between the HTP and LTP studies remained at only ∼3% of the total unique sites in version 2.0 of the database (Table 1). The Phospho.ELM database also records phosphorylation sites from both LTP and HTP studies, although without a focus on an individual species. Interestingly, the most recent update of Phospho.ELM (version 9.0) reported >45 000 unique sites, but with only 846 sites in common between LTP and HTP data sets, which represents ∼2% of the total unique sites (34). To date, the incidence of sites discovered by LTP studies relative to HTP approaches appears similar across species, and while constrained by the amount of LTP data, the limited overlap poses a conundrum in the interpretation of HTP data.

**Figure 1.**
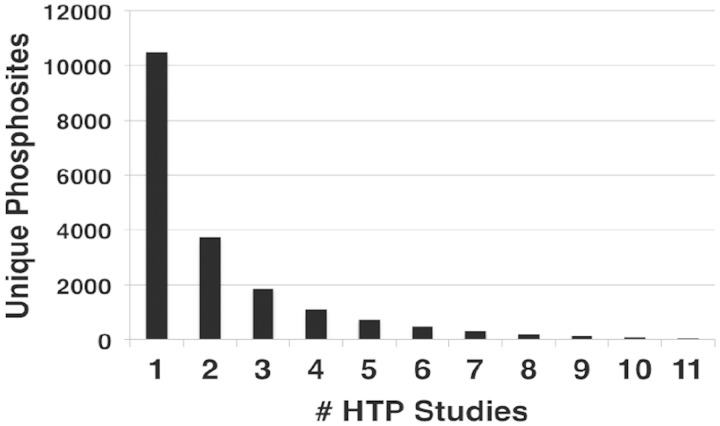
Number of occurrences of unique phosphorylation sites in different HTP studies. The number of unique sites identified in the indicated number of different HTP studies.

Within the LTP data set, phosphorylation of a significant number of residues have been confirmed by multiple experimental techniques, represented by different evidence codes (Table 2). A summary of the number of different evidence codes for each unique site in PhosphoGRID is provided in Supplementary Table S1. Phosphorylated residues that were demonstrated by five or more different techniques are detailed in Table 3. Typically, these residues were initially identified in LTP studies as phosphosites because of effects on protein activity, and accordingly 91% have a defined function annotated in PhosphoGRID (61 out of 67 total). In many cases, the presence of phosphorylation was subsequently confirmed by liquid chromatography (LC)-MS of phosphopeptides recovered from purified proteins, such that nearly one-half of sites described in LTP studies (794, Table 2) were initially detected, or were confirmed by MS approaches. It is also becoming increasingly common for investigators to produce specific anti-phosphopeptide antibodies against the phosphorylated residue(s), which provides additional confirmation of the phoshosphorylation, and also allows for simplified detection of the phosphorylation site under various experimental conditions (Tables 2 and 3). It is interesting to note that 76% of phosphosites supported by at least five different techniques in the LTP data set were also present in one or more HTP data sets (45 of 59 sites). In fact, 35 of these well-characterized sites were discovered in at least two HTP studies, which are significant because ∼11 000 unique sites were represented in only one HTP study, whereas ∼3000 sites were found in four or more HTP studies (Figure 1). In total, 731 phosphorylated residues found in LTP studies were discovered in two or more HTP data sets, which represents 45% of the total LTP sites. Thus, although overlap between the LTP and HTP phosphosites within the entire data set is limited (Table 1), there is substantial overlap for sites that have been most thoroughly characterized.

**Table 2.** Summary of techniques providing evidence for specific phosphosites in LTP studies

Technique	Residues^a^	Proteins^b^
MS analysis of phosphopeptides^c^	794	122
Phosphopeptide fingerprint	78	39
Shift in protien mobility on SDS–PAGE	612	114
Loss of 32P label from protien with a mutation	159	60
Recognized by specific anti-phosphopeptide antibody	180	54
Recognized by anti-pT/pS antibody	41	40
Recognized by anti-pY antibody	5	4
Mutation of the residue affects activity	902	224
Phosphorylation of a synthetic peptide bearing the residue	180	21
Identity to known phosphorylation on species ortholog	116	33
Loss of phosphorylation *in vitro* with mutation of the residue	322	103
D or E substitution enhances activity of the protein	193	61
Edman degradation of peptide	17	10
Loss of isoelectric isoform with a mutation of the residue	42	18
Detected by Phos-Tag or pro-diamond Q phospho-specific stain	17	9

^a^Number of individual phosphorylated residues detected by the indicated technique in LTP studies.

^b^Number of proteins analyzed by the indicated technique in LTP studies.

^c^Phosphosites identified using MS sequencing or mass determination of phosphopeptides.

**Table 3.** Phosphorylation sites demonstrated by five or more experimental techniques

Protein	Residue(s)	Codes^a^	MS^b^	APP^c^	Activity^d^	HTP^e^
Fbp1	S12	7	Yes	No	Yes	1
Fus3	Y182	7	Yes	Yes	Yes	3
Gal4	S699	7	Yes	Yes	Yes	1
Hxk2	S15	7	Yes	No	ND	6
Sic1	S201	7	Yes	Yes	Yes	3
Cdc13	S306	6	Yes	Yes	ND	1
Cki1	S85	6	Yes	No	Yes	3
Fus3	T180	6	Yes	Yes	Yes	1
Hog1	T174, Y176	6	Yes	Yes	Yes	3, 6
Rfa1	S178	6	Yes	No	Yes	9
Sic1	T173	6	Yes	No	Yes	1
Spc110	S60	6	Yes	No	ND	6
Sui2	S52	6	No	Yes	Yes	0
Ace2	S122	5	Yes	Yes	Yes	3
Atg1	T226	5	Yes	Yes	Yes	0
Cbk1	T743	5	No	Yes	Yes	0
Cdc19	S22	5	Yes	No	Yes	6
Cdc28	T169	5	Yes	Yes	Yes	4
Cdh1	S42	5	No	No	Yes	0
Cdh1	T157	5	Yes	No	ND	1
Cki1	S25, S30	5	No	No	Yes	0
Dam1	S257, S265, S292	5	Yes	Yes	Yes	2, 1, 2
Gsy2	S655	5	Yes	No	Yes	7
Hta1	S129	5	Yes	Yes	Yes	3
Ime2	Y244	5	Yes	No	Yes	0
Kss1	T183, Y185	5	Yes	Yes	Yes	1, 2
Maf1	S90, S177, S178, S209, S210	5	Yes	Yes	Yes	3, 3, 2, 3, 2
Mig1	S278, S381	5	Yes	No	Yes	4, 1
Nab2	T178, S180	5	Yes	No	No	0
Npl3	S411	5	Yes	No	Yes	0
Pah1	S110, S114, S723	5	Yes	No	Yes	0
Pah1	S168, S744, S748	5	Yes	No	Yes	2, 7, 8
Pho4	S100	5	Yes	No	Yes	0
Pho4	S152, S223	5	Yes	No	Yes	5, 2
Rpp1B	S96	5	Yes	No	Yes	4
Sch9	S711, T737, S758, S765	5	Yes	No	Yes	0
Sch9	S723, S726	5	Yes	No	Yes	4, 8
Shs1	S519, S541	5	Yes	No	Yes	7, 5
Sko1	S108, T113	5	Yes	No	Yes	1, 2
Spc110	T64, T68	5	No	No	No	0
Sso1	S79	5	Yes	Yes	Yes	0
Swi6	S160	5	Yes	No	Yes	4
Ura7	S424	5	Yes	No	Yes	0
Ycf1	S251	5	Yes	Yes	Yes	7

^a^Number of different evidence codes for the phosphorylation.

^b^The phosphorylation was detected by LC-MS on purified protein (Yes/No).

^c^The phosphorylation was detected by specific anti-phosphopeptide antibodies (Yes/No, APP = antiphosphopeptide antibody).

^d^Mutation of the residue affects activity of the protein (Yes/No, ND = not determined).

^e^The number of independent HTP studies where the specific phosphorylation was discovered.

## Extent of the phosphoproteome

**Figure 2.**
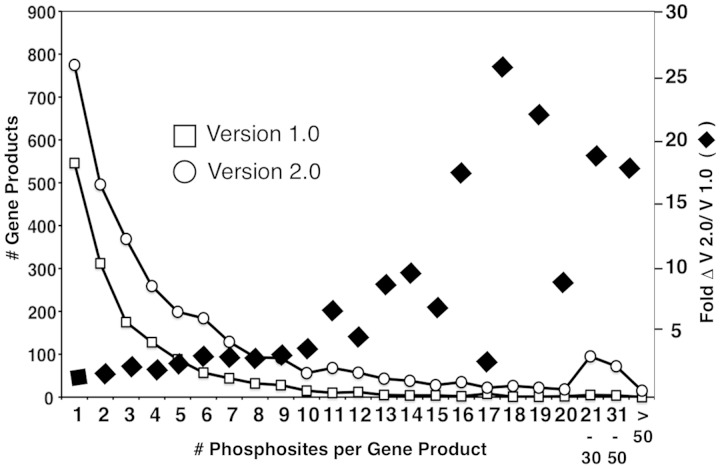
Distribution of multiply phosphorylated proteins in PhosphoGRID versions 1.0 and 2.0. Proteins with the indicated number of unique phosphorylated residues in PhosphoGRID versions 1.0 (open box) and 2.0 (open circle). The fold change in the number of phosphosites per protein between V 1.0 and 2.0 are indicated by black diamonds.

A primary goal of MS-based phosphoproteomics efforts is to generate a complete catalog of relevant phosphorylated proteins and specific phosphorylation sites, and to determine how these change in response to environmental and developmental conditions. The expectation is that these resources will enable implementation of novel bioinformatic strategies for analysis and prediction of protein kinase/phosphatase-substrate relationships and the elaboration of signaling networks. The version 2.0 PhosphoGRID update has added an additional ∼15 000 sites from HTP studies, a 4-fold increase from version 1.0 (Table 1). Given that the number of total phosphoproteins has only increased by 2-fold (Table 1), the majority of new unique sites occur on previously documented phosphoproteins. Concordantly, the number of proteins with a single phosphorylation site increased from 556 to 775, i.e. only a 1.4-fold increase (Figure 2). Furthermore, most of the new unique phosphorylations from HTP studies were documented on already known multiply phosphorylated proteins (Figure 2). A recent analysis of a high confidence merged data set representing 12 independent HTP studies, called the 12 HQ data set, suggested that the yeast phosphoproteome is gradually approaching saturation because the average overlap of phosphosites between any two experiments is 12%, whereas the overlap between phosphoproteins in total is 28% (35). Similarly, from comparisons of overlap between different HTP studies, it was estimated that detection of the yeast phosphoproteome has reached 80–90% coverage (36). Given the ∼3200 phosphoproteins documented in PhosphoGRID 2.0, these estimates suggest that 3800 phosphoproteins may be present in yeast under standard growth conditions, corresponding to approximately two-thirds of the entire proteome. It should be noted that, unlike the 12 HQ data set (35), we have included entries in PhosphoGRID of every phosphorylation site defined by a cut-off score designated as significant by authors of the HTP manuscripts. However, for each site from HTP studies, we plan to incorporate the published confidence scores in future updates.

## Most phosphosites identified in HTP studies are uncharacterized

The full exploitation of HTP studies will require functional characterization of many more phosphosites to chart the phosphorylation-based regulatory events that control cellular behavior. PhosphoGRID documents the function of specific phosphorylated residues, including both specific biochemical effects and general phenotypic consequences. Not surprisingly, the majority (∼60%) of phosphosites characterized in LTP studies have an assigned function, often deduced from a mutant phenotype (Table 3). Annotation of LTP articles for the version 2.0 update added an additional 249 unique sites on 25 proteins with defined function (Table 4).

**Table 4.** Defined functions for phosphorylation sites in PhosphoGRID 2.0

Effect on Function	Residues^a^	Proteins^b^
Specific effects on protein structure/function		
Promotes a protein interaction	222	42
Inhibits a protein interaction	212	35
Modifies interaction with small molecule/ligand	11	7
Functional consequence of phosphorylation		
Activates protein function	240	88
Inhibits protein function	124	27
Targets protein for degradation	92	24
Enhances protein stability	30	6
Modifies subcellular localization	126	51
Total	940	225
Increase from version 1.0	249	25

^a^Number of individual phosphorylated residues in PhospoGRID assigned the indicated function.

^b^Number of proteins in PhosphoGRID bearing a phosphoresidue assigned the indicated function.

**Figure 3.**
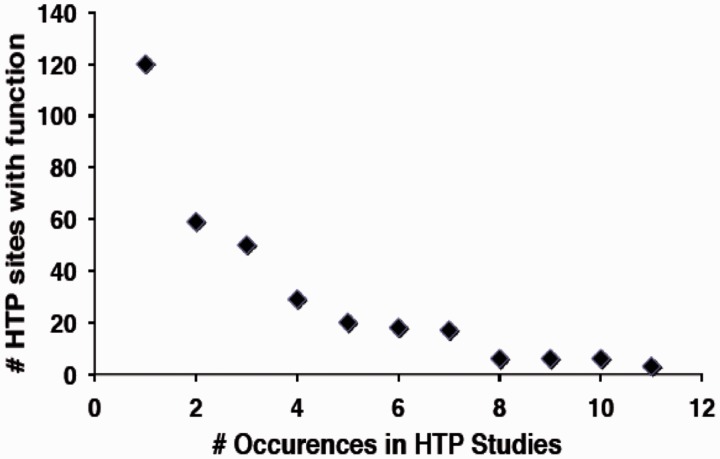
Relationship between the number of sites with characterized function and their frequency of occurrence in HTP studies.

In contrast, a much smaller fraction of sites identified in HTP studies (∼3%) have a defined function by virtue of overlap with the LTP data set (Table 1). Based on analysis of the 12HQ data set, is was suggested that sites identified three or more times in HTP studies are most likely to represent regulatory phosphorylation events, rather than inconsequential or non-specific phosphorylation (35). By application of this criterion to the version 2.0 data set, ∼4900 phosphorylated residues on 1500 proteins in the yeast proteome might confer an obvious regulatory function. However, because of the stringency applied to assemble the 12HQ data set, this is probably a conservative estimate (35). We examined whether there was a correlation between the number of occurrences of specific phosphoresidues in the HTP data sets and how many of those were shown to confer function in LTP studies. We in fact observed a strong negative correlation between the number of sites identified in HTP sites with function and the total number of occurrences in different HTP studies (Figure 3), suggesting that sites identified in multiple HTP studies are less well characterized for function.

Several factors likely contribute to the low representation of characterized sites in HTP data. First, HTP data sets are enriched in phosphopeptides from proteins of higher relative abundance (35, 36). We observe that phosphosites identified in LTP studies derive from proteins of slightly lower average abundance (∼1600 molecules per cell, median 1800) than phosphosites identified in HTP experiments (∼1800 molecules per cell, median 1800), but this difference may be an underestimate because only 70% of proteins in the LTP data set have associated abundance measurements (37). Protein phosphorylation is correlated with a shorter protein half-life (38), and proteins within the 12HQ data set were found to have ∼50% shorter half-life than presumptive non-phosphoproteins not present in the data set (35). Considering that at least 10% of defined phosphorylations target proteins for degradation (Table 4), the most unstable phosphoproteins are unlikely to be adequately represented in proteomic data sets measuring protein stability. Consequently, the 10% of phosphorylations shown to promote degradation in LTP studies (Table 4), is likely a substantial underrepresentation because proteins with half-lives of <10 minutes can be difficult to detect by MS methods (39). Finally, and perhaps most importantly, the sparse nature of the current LTP data set inherently limits its applicability as a benchmark to evaluate sites identified in HTP studies. For example, there are 404 residues in PhosphoGRID that have been discovered eight or more times in separate HTP studies (Supplementary Table S2), and of these, only 15 have been examined for function (Table 5). Overall, at present, the frequency of phosphosite discovery is not obviously predictive of function. Consequently, bioinformatic prediction of functional phosphorylated residues will require utilization of additional parameters that may include, but not limited to, protein kinase consensus sequences, subcellular distribution, evolutionary conservation and polymorphic variability (35). PhosphoGRID will become increasingly useful for this purpose as the data sets, and linkage of specific sites to protein kinases, phosphatases, physiological conditions and functions is expanded.

**Table 5.** Functional sites reported eight or more times in independent HTP studies

Gene	HAA	R#	HTP	Function
*GSY2*	S	651	11	Inhibits Gsy2 function
*MYO5*	S	357	11	Activates Myo5 function, required to sustain polarized actin cytoskeleton
*UGP1*	S	11	11	Regulates Ugp1 conformational state
*BNI5*	S	346, 350	10	Required for interaction of Bni5 with Cdc11
*CDC37*	S	14	10	Activates Cdc37 function, mutation produces sensitivity to cell wall stress (note sometimes referred to as S13)
*NAP1*	S	177	10	Phosphorylation of Nap1 by CK2 regulates nuclear import
*RPP2B*	S	100	10	Targets Rpp2B for degradation
*SHS1*	S	447	10	Regulates Shs1 interaction with Gin4, phosphorylated by Pho85
*CDC28*	Y	19	9	Inhibits Cdc28 function
*CDC3*	S	509	9	Promotes dissasembly of obsolete septin ring from the previous cell cycle
*PMA1*	T	912	9	Required for Pma1 function, Tor/Sch9-dependent
*RFA1*	S	178	9	Required for Rfa1 interaction with Mec1
*SCH9*	S	726	9	Required for Sch9 function, phosphorylated by Tor1
*CDC37*	S	17	8	Required for Cdc37, phosphoryated by CK2
*SHS1*	S	447	8	Regulates Shs1 interaction with Gin4, phosphorylated by Pho85
*CHO1*	S	46	8	Required for Cho1 function, phosphorylated by TPK1
*GSY2*	S	655	8	Inhibits Gsy2 function
*PAH1*	S	748	8	Inhibits protein Pah1 function, mutation in combination with other S/T residues causes an increase in specific phosphatidic acid phosphatase activity
*SHS1*	T	539	8	Regulates Shs1 interaction with Gin4
*WHI5*	S	62	8	Mutation (along with other Cdk8 consensus sites) causes synthetic lethality with Swi6-4SA

HAA = hydroxylamino acid, R# = residue number

## Phosphorylation-based signaling networks

An important feature of PhosphoGRID is the documentation of experimental evidence for phosphorylation/dephosphorylation of specific sites by protein kinases and phosphatases, respectively, as well as the involvement of regulatory subunits. PhosphoGRID also records the specific conditions under which phosphorylation events occur. These details will facilitate the predicting and mapping of complex signal transduction networks (40). The version 2.0 update contains a significant expansion of phosphosites that occur under specific physiological conditions, typically indentified by the differential SILAC labeling technique (41). In total, these studies have expanded the number of unique phosphosites that appear under specific conditions by nearly 6-fold to ∼3200 sites on ∼1100 proteins. Examples include the response to the target of rapamycin (TOR) inhibitor rapamycin (10, 11), osmotic stress (14) and DNA damage (15). A MS-based study with a chemically conditional analog-sensitive allele of *CDK1*/*CDC28*, resulted in the identification of >2000 cell cycle–regulated phosphorylation events on ∼800 proteins (12). These HTP studies also added evidence for phosphorylation by specific protein kinases, typically based on a genetic requirement for the phosphorylation *in vivo* in combination with a match of the site to a well-defined consensus sequence. These new data include ∼700 sites for Cdk1/Cdc28, ∼160 sites for Mec1/Tel1 and ∼150 sites for Rad53 during DNA damage response (15), and 46 rapamycin-sensitive sites specific for Tor1 (10, 11). Additionally, we have documented 456 sites whose abundance decreases 4-fold or more in strains deleted of genes encoding protein kinases (17). Interestingly, and important to note, deletion of genes encoding both kinases and phosphatases typically results in both decreases and increases in the abundance of a large number of specific phosphorylations, which demonstrates the interconnection of global phosphorylation networks (17). Consequently, it is becoming increasingly clear that most, if not all, protein kinases and phosphatases operate within networks that include multiple inputs by upstream regulators as well as negative feedback loops (13, 42). For each phosphosite where abundance is altered in strains deleted of protein kinase or phosphatase genes, we have documented the relative fold change in the ‘Notes’ field for the specific residue (17).

In total, with the version 2.0 update, we have documented protein kinases for 2257 phosphosites, representing ∼10% of the total unique sites, 482 of which also have documented evidence for the involvement of specific cyclins or other regulatory subunits. In these instances for the MS proteomics studies, evidence of phosphorylation by specific protein kinases typically includes a genetic requirement for the phosphorylation *in vivo* in combination with a match of the site to a well-defined consensus sequence. Ideally, however, definitive proof that specific sites are phosphorylated by a protein kinase requires demonstration that the enzyme is capable of phosphorylating purified recombinant protein substrate on the same residue *in vitro*.

Regulation of protein function by dephosphorylation is an important but still underappreciated component of signal transduction networks (43). In contrast to the rapid expansion of phosphosites associated with specific protein kinases, far fewer sites are described as substrates for protein phosphatases. In PhosphoGRID version 2.0, only 259 sites on 102 proteins are associated with specific phosphatases, and of these, only 19 implicate a specific phosphatase regulatory subunit. Approximately one-half of these are represented by sites whose abundance was increased 4-fold or more in strains deleted of genes encoding phosphatase catalytic subunits (17), while the remainder were identified in LTP studies focused on regulation of specific proteins by dephoshorylation. The identification and characterization of phosphatases that remove specific phosphorylated residues is inherently more difficult than analysis of kinase-mediated phosphorylation, and we expect that the number of sites associated with specific phosphatases will expand considerably with additional large-scale MS-based efforts in the near future.

Decades of research have entrenched the concept that protein kinases and phosphatases do not act in isolation but instead form complex networks, in which each enzyme regulates one or more other enzymes, often accompanied by embedded positive and negative feedback loops (40). These features can in part be inferred from phosphorylation sites that are identified on the kinases and phosphatases themselves, in either LTP or HTP studies. A recent systematic analysis of the kinase/phosphatase interaction network of yeast revealed 485 phosphorylation sites on kinases and phosphatases, as well as on their interaction partners (13). In total, the version 2.0 data set contains 1506 documented sites on protein kinases, 94 sites on protein phosphatases and 149 sites on kinase or phosphatase-associated regulatory subunits. The integration of interaction data and phosphorylation site data across the entire PhosphoGRID data set should facilitate the prediction of new network elements and information flow in the form of regulated phosphorylation events.

## Conclusions and perspective

The PhosphoGRID version 2.0 update represents a substantial expansion of the phosphosite data set, as derived from both HTP and LTP studies, which to date have surprisingly little overlap. Our analysis, and that of others (35), indicates that discovery of phosphorylated proteins in the yeast proteome is becoming saturated, but that the number of unique phosphorylated residues continues to grow with each HTP study reported. Based on the current PhosphoGRID HTP data set, it does not appear possible to predict the relevance of specific phosphorylated residues based on frequency of discovery alone. Aside from the putative roles of particular individual sites, this issue will be critical to understand the function and evolution of the phosphoproteome. From an evolutionary perspective, it has been argued that the rapid evolution of much of the phosphoproteome arises because a large fraction of the sites are non-functional and thus not subject to selective pressure (44). This notion raises the important issue of how to define phosphosite function, especially because many proteins are phosphorylated on multiple sites that individually may be dispensable but collectively may confer an important function, such as cooperative protein recognition or regulation (45–50). The continued accumulation of evidence for phosphosite function based on LTP data in PhosphoGRID will allow this and other hypotheses regarding the phosphoproteome to be tested systematically.

## Supplementary data

Supplementary data are available at *Database* Online.

## Funding

This work was supported by Grant Number 0011258 from the Canadian Cancer Society Research Institute (IS), Grant Numbers 1R01RR024031-01 and R24RR032659 from the NIH National Center For Research Resources (M.T., K.D.), Grant Number BB/F010486/1 from the Biotechnology and Biological Sciences Research Council (M.T.) and Grant Number HOP-120237 from the Canadian Institutes for Health Research (I.S.). Funding for open access charge: Canadian Institutes for Health Research (I.S.) Grant Number HOP-120237.

*Conflict of interest*. None declared.

## Supplementary Material

Supplementary Data
