# Supplementary material for: The PhosphoGRID Saccharomyces cerevisiae protein phosphorylation site database: version 2.0 update
Source: Database (Oxford). 2013 May 11;2013:bat026. doi: 10.1093/database/bat026 (PMC3653121; doi:10.1093/database/bat026)
Supplement: Supplementary Data [file supp_2013_bat026_index.html]

The PhosphoGRID Saccharomyces cerevisiae protein phosphorylation site database: version 2.0 update — Supplementary Data 

# The PhosphoGRID *Saccharomyces cerevisiae* protein phosphorylation site database: version 2.0 update

## Supplementary Data

files

**Files in this Data Supplement:**

- Supplementary Data - xls file
- Supplementary Data - xls file
